# The People

**Published:** 1889-04-27

**Authors:** 


### The People.

What are we to understand by "the people"? From the point of view of our present purpose "the people" may be regarded as all those who have at any time required, or who may in the future require, charitable aid. Help in sickness is one of the gains of a developed humanity. It is not natural to man, as man, to be exceedingly regardful of the powerless. To fear the cruel, to stand in awe of the powerful, to flatter those who have benefits to bestow—these are the natural and common ways of men. But to pity the suffering because he is suffering; to help the poor and the helpless because they are poor and helpless—this is distinctly a resultant of long ages of educational development on upward and noble lines. The gain to the poor is incalculable. But any, even the richest man, may by some unexpected turn of fortune become both poor and helpless before the battle of life is fought out to its final issue. Therefore the gain to the whole world resulting from the present tone and temper of civilised society is also immeasurable.

The common advantages which the people enjoy in this country are not highly valued, for the simple reason that they are common. They are taken as a matter of course. The disadvantages which are peculiar to a highly-developed civilisation are marked with microscopic scrutiny, and often magnified by microscopic powers. No wise man, much less a philanthropist, desires to blind the people to any disadvantages under which they may live; rather, he will lend a hand to their removal. But every man of any mental capacity at all ought to wish to take a just view of his position, and to see its advantages as clearly as its disadvantages. It has been somewhere recorded that a young man who had a modest but sure income once wrote to Mr. Spurgeon, asking for advice as to how he might improve his position. The practical preacher promptly advised the young man never to get off the back of one horse until he had a better ready to mount. Homely advice, undoubtedly; but does the whole region of philosophy or of common sense suggest any better answer which might have been given?

It is not a mark of good sense for a man to estimate wrongly his own position, and it is not an indication of a wholesome disposition to undervalue benefits received. In this country there are drawbacks, certainly. But is there any country in which there are none? Perhaps, all things considered, we can compare very favourably with any other country in the matter of drawbacks. As for advantages, there are sensible persons who think that in certain departments of life the people have too many. This is particularly the case with regard to charitable aids. Mr. Booth has recently published facts and statistics which seem to show that increased charity is in many instances the direct cause of increased pauperism. The careful enquiry lately made into the condition of the workers of Central London entirely confirms that view. It is of no use shutting our eyes to bad facts. There are certain human creatures whose passion is idleness and whose vice is parasitism. They will not work except on compulsion; and they will live—but at other people's expense. These creatures belong principally to the working classes. It is the duty and the interest of the honest workers to purge themselves of the suspicion of idleness and shiftlessness. The pride and the manhood of the workman should make him insist that so far as it is in his power he will raise his class to an equality with every other class in everything that constitutes true worth and human value. We know well that there are worthless workmen who will sneer at this as the utterance of one who knows nothing of the facts of the case. But the plain truth is this, that a large proportion of working men in every town and village are self-respecting, honest, and honoured men, whose character will bear comparison with that of the very worthiest and noblest of their neighbours. If the attainment of such a position and character is possible for some, why not for all? The real strength, indeed, of the working class depends upon the force and energy with which they devote themselves to an honest and virtuous life. No power on earth can bend the British workman down if he will be true to the highest instincts and the worthiest habits of his fellow-countryman.

It is to all classes of Englishmen a pride and a delight to see everywhere the gentle form of charity gliding in and out and ministering tenderly to all the wants of the people. That is what the philanthropist loves, and from his point of view, when that is done efficiently, all is as it should be. But the man who deeply and sincerely respects his fellow-countrymen of every class desires to see them not merely ministered to but ministering; not waiting till others come to their help, but standing firmly on the basis of their own manhood and going forth like a valiant army to help others. A city of beggars! What a terrible sight! A nation of paupers! What a shameful destiny! Charity let us have, free, generous, abundant for the helpless, the young and the old, by all means; but in the name of all that is manly, all that is patriotic, all that is best in the present and noblest in the past of British history, let us have manly independence and self-help to the very last breath.

